# P-1449. An Educational Initiative to Improve Engagement in HIV Services within Substance Use Disorder Clinics

**DOI:** 10.1093/ofid/ofae631.1621

**Published:** 2025-01-29

**Authors:** Chad J Zawitz, Kelly E Pillinger, Shaina Y Vincent, Laura Simone, Chris Napolitan, Jeffrey D Carter, Jenniffer A Meza Jimenez, Leah Molloy

**Affiliations:** John H Stroger, Jr Hospital of Cook County and CORE Center, Chicago, Illinois; PRIME Education, New York, New York; PRIME Education, New York, New York; PRIME Education, LLC, Fort Lauderdale, Florida; PRIME Education, New York, New York; PRIME Education, LLC, Fort Lauderdale, Florida; PRIME Education, New York, New York; PRIME Education, New York, New York

## Abstract

**Background:**

Substance use disorder (SUD) clinics are uniquely positioned to link patients with SUD to HIV services. This project aimed to bridge gaps in HIV care within SUD clinics.

**Methods:**

From 6/2023-10/2023, healthcare professionals (HCPs) led 5 education sessions on HIV testing, prevention, and management at SUD clinics in West Virginia and Delaware. Surveys were administered pre- and post-sessions and again 4-6 weeks later. Results informed a workshop for HCPs and policy makers and an online toolkit for HCPs nationwide.

**Results:**

Surveys were completed by 67 SUD clinic patients (mean age: 48 years; 66% female; 76% Caucasian; 61% previously incarcerated; 78% previously tested for HIV, 7% living with HIV) and 35 HCPs. After the sessions, significant improvements were seen in patient knowledge of “undetectable = untransmittable” (27% vs 63%; P < .001), PrEP awareness (62% vs 88%; P = .001), SUD support resources (73% vs 91%; P = .008) and HIV care resources (43% vs 70%; P = .003). Pre-session, 64% of patients did not perceive the need for HIV testing. Of the patients who did (n = 24), anxiety (63%) was the top barrier, while HCPs perceived it was patients’ lack of HIV awareness (63%). Regarding PrEP, HCPs most frequently perceived keeping up with appointments (54%) and remembering doses (51%) as the greatest challenges their patients face, but patients more frequently identified not knowing enough about the medication (39%) and concerns about side effects (31%). Patients’ self-reported likelihood of getting tested for HIV was 18% before the education and was 28% afterwards (P = .147), and 1 month later participating HCPs reported having helped > 250 patients with SUD get HIV testing. On follow-up, HCPs also described continued use of the education materials with 80% planning to lead another group session. After attending the live workshop, HCPs and policy makers (N = 103) committed to improving HIV services for patients with SUD with 77% identifying single site services for both HIV and SUD care as a high priority. As of 3/2024, the online toolkit was accessed by 1,347 HCPs.

**Conclusion:**

Patient knowledge of HIV testing, prevention, and management increased following the program which also offered insights into community-level awareness and barriers to engaging in HIV services.

**Disclosures:**

**Chad J. Zawitz, MD**, Gilead Sciences: Honoraria **Kelly E. Pillinger, PharmD**, AHFS: Contractor

